# Supplementary material for: High-confidence 3D template matching for cryo-electron tomography
Source: Nat Commun. 2024 May 11;15:3992. doi: 10.1038/s41467-024-47839-8 (PMC11088655; doi:10.1038/s41467-024-47839-8)
Supplement: Supplementary file 1 — Supplementary Information [file 41467_2024_47839_MOESM1_ESM.pdf]

High-confidence 3D template matching for cryo-electron tomography

Sergio Cruz-León[1], Tomáš Majtner[2], Patrick C. Hoffmann[2], Jan Philipp Kreysing[2,3], Sebastian Kehl[4], Maarten W. Tuijtel[2], Stefan L. Schaefer[1], Katharina Geißler[2,3], Martin Beck[2,5*], Beata Turoňová[2,*] & Gerhard Hummer[1,6,*]

[1]Department of Theoretical Biophysics, Max Planck Institute of Biophysics, Max-von-Laue-Str. 3, 60438 Frankfurt am Main, Germany
[2]Department of Molecular Sociology, Max Planck Institute of Biophysics, Max-von-Laue-Str. 3, 60438 Frankfurt am Main, Germany
[3]IMPRS on Cellular Biophysics, Max-von-Laue-Str. 3, 60438 Frankfurt am Main, Germany
[4]Max Planck Computing and Data Facility, Gießenbachstraße 2, 85748 Garching, Germany
[5]Institute of Biochemistry, Goethe University Frankfurt, 60438 Frankfurt am Main, Germany
[6]Institute of Biophysics, Goethe University Frankfurt, 60438 Frankfurt am Main, Germany

E-mail: martin.beck@biophys.mpg.de; beata.turonova@biophys.mpg.de; gerhard.hummer@biophys.mpg.de

Supplementary Figures

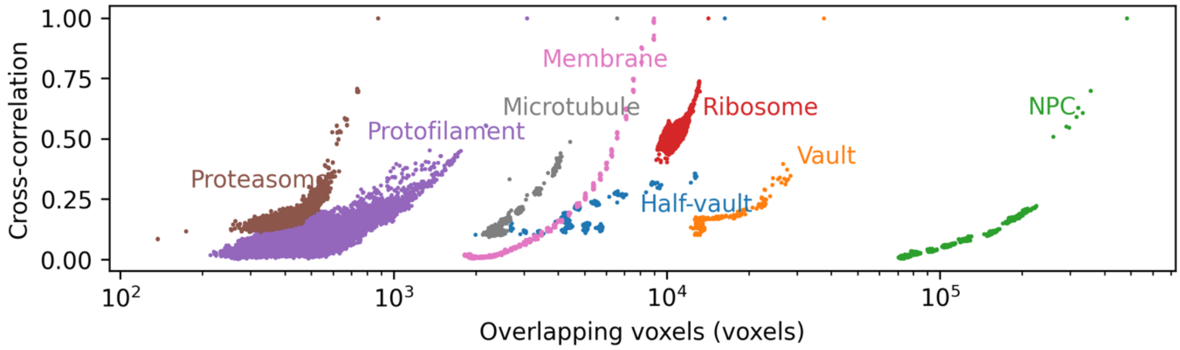

Supplementary Fig. 1: Cross correlation as a function of the number of overlapping voxels. Scatter plot of the dependence of the constrained cross-correlation of a template with itself (*in silico* evaluation) as a function of the number of overlapping voxels. All the calculations were done for a pixel size of 8.7 (Å) and an angular step of 10 deg. Each plotted point corresponds to a possible rotation.

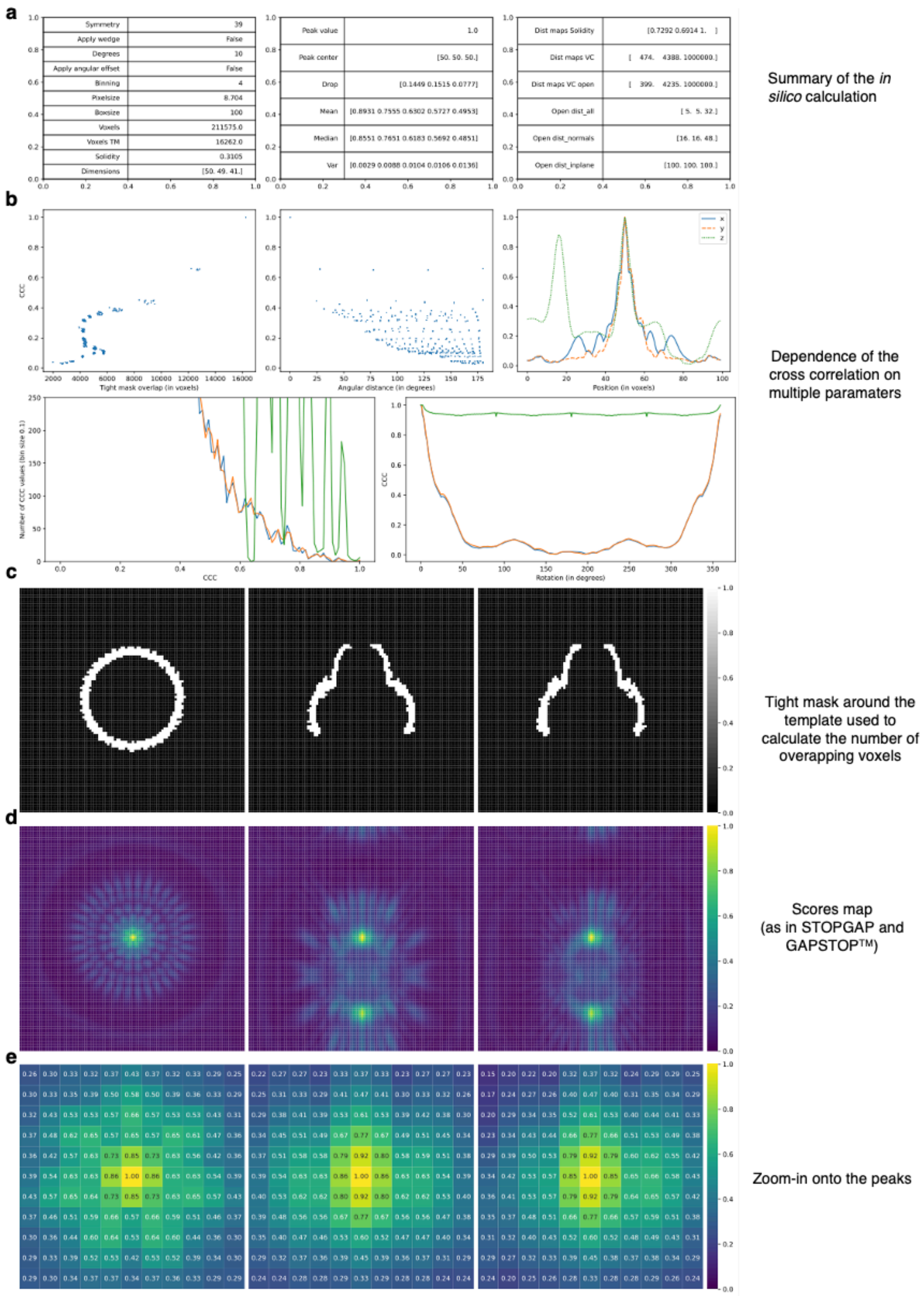

Supplementary Fig. 2: Example output of the *in silico* assessment of cross-correlation (half vault). a, Summary of the input parameters and the main outputs of the *in silico* calculation. **b**, Constrained cross correlation (CCC) as a function of the number of overlapping voxels (tight mask overlap), angular distance, position and rotations. **c**, Tight mask around the template. For the vault, we used its two halves as template, resulting in two peaks along the z-direction. **d**, Scores-map (as in STOPGAP and GAPSTOP^{TM}) with a zoom-in onto the peaks (**e**).

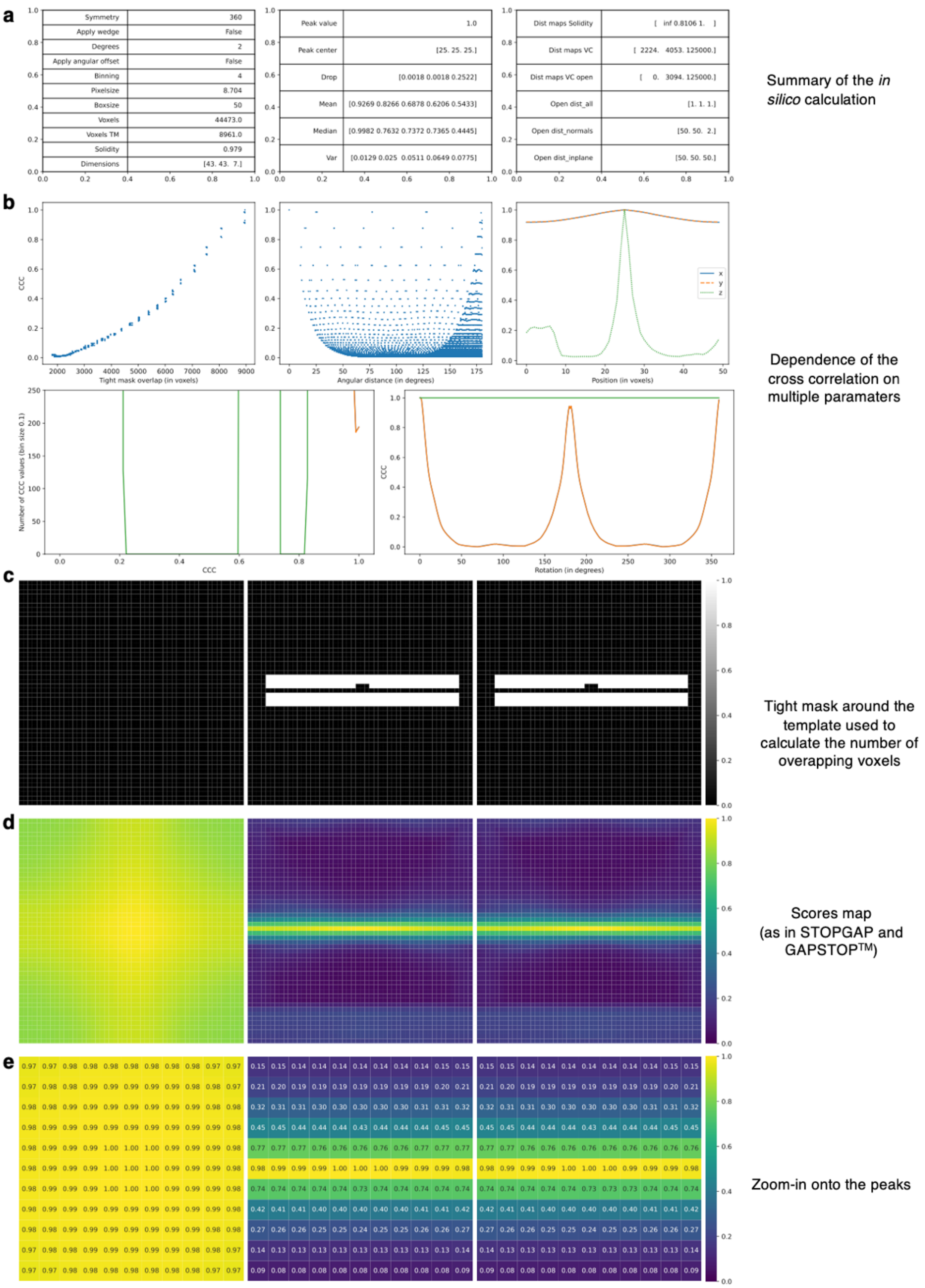

Supplementary Fig. 3: Example output of the *in silico* assessment of cross-correlation (Membrane - small STA). a, Summary of the input parameters and the main outputs of the *in silico* calculation. **b**, Constrained cross correlation (CCC) as a function of the number of overlapping voxels (tight mask overlap), angular distance, the position and rotations. **c**, Tight mask around the template. **d**, Scores-map (as in STOPGAP and GAPSTOP^{TM}) with a zoom-in onto the peaks (**e**). The CC scores map revealed a peak that extends into the x-y plane and was unaffected by rotation in the x-y plane, consistent with the symmetry of the membrane.

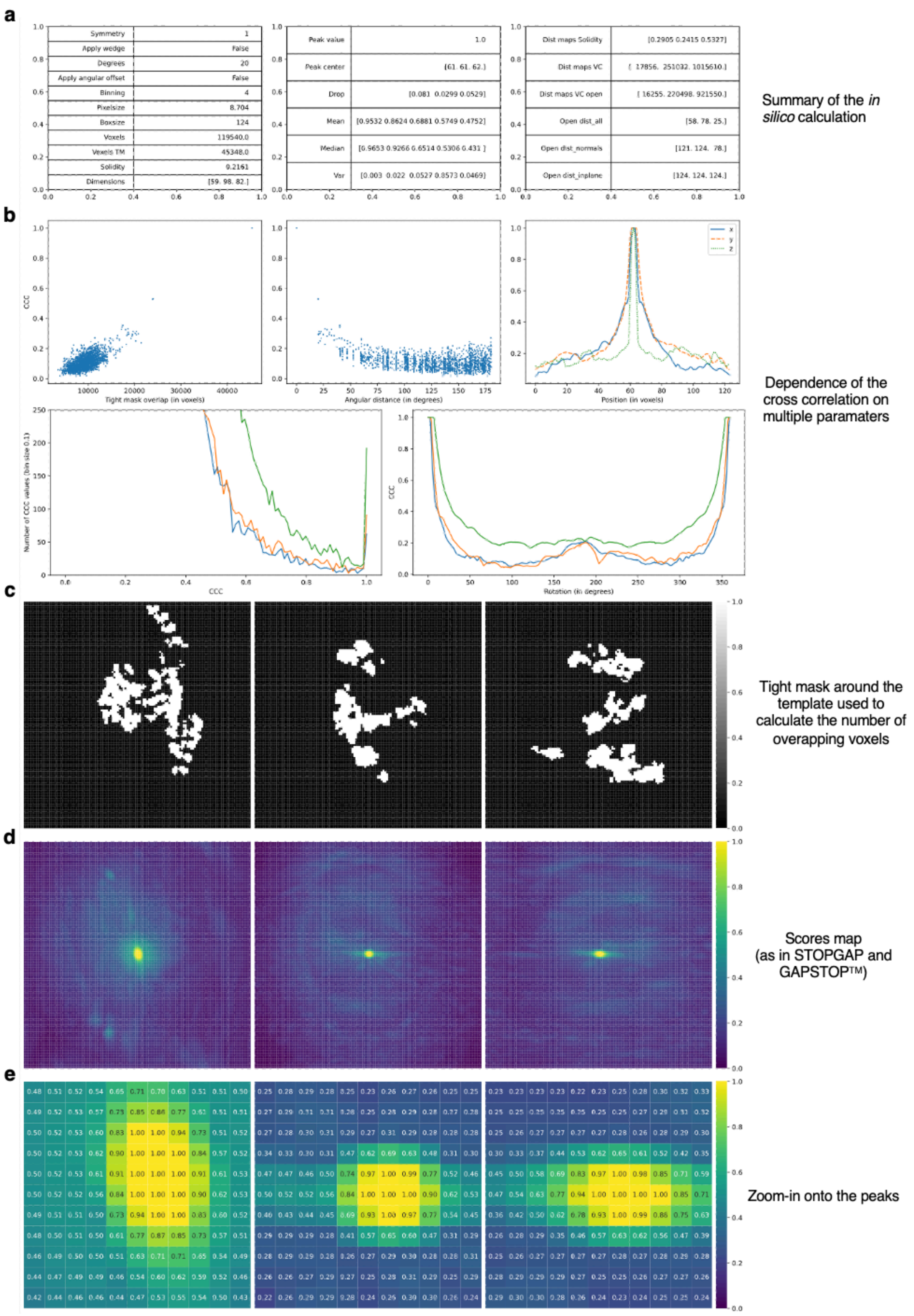

Supplementary Fig. 4: Example output of the *in silico* assessment of cross-correlation (NPC subunit without membrane). **a**, Summary of the input parameters and the main outputs of the *in silico* calculation. **b**, Constrained cross correlation (CCC) as a function of the number of overlapping voxels (tight mask overlap), angular distance, position and rotations. **c**, Tight mask around the template. For the vault, we used its two halves as template, resulting in two peaks along the z-direction. **d**, Scores-map (as in STOPGAP and GAPSTOP^{TM}) with a zoom-in onto the peaks (**e**).

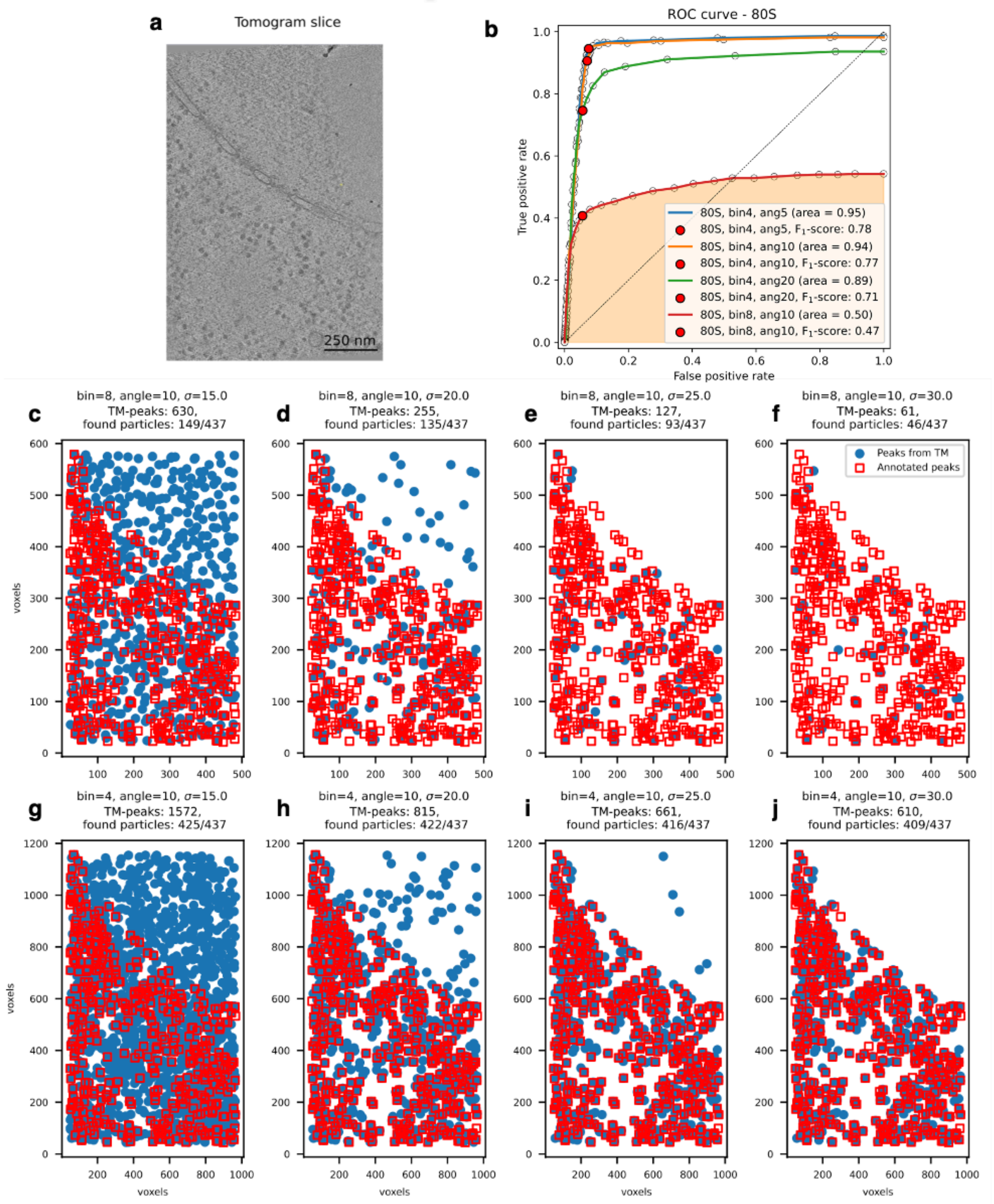

Supplementary Fig. 5: Template matching results for the 80S ribosome with 8-binned data (bin-8: 17.40 Å/voxel, top row) and 4-binned data (bin-4 of 8.704 Å/voxel, bottom row). a, Tomogram slice. **b**, ROC curves, area under the curve, and maximum F_1-score for the 80S at bin4 at different angle steps and bin8 with an angle step of 10 deg. **c**-**j**, Superimposition of the peaks obtained from template matching (blue circles; sampled every 10 degrees with varying cross-correlation thresholds) and the high-confidence localizations obtained from an expert multiple-step alignment using Relion[1] as described in reference [2]. The number of peaks as well as the ratio of "TM-found" particles is described in the figure.

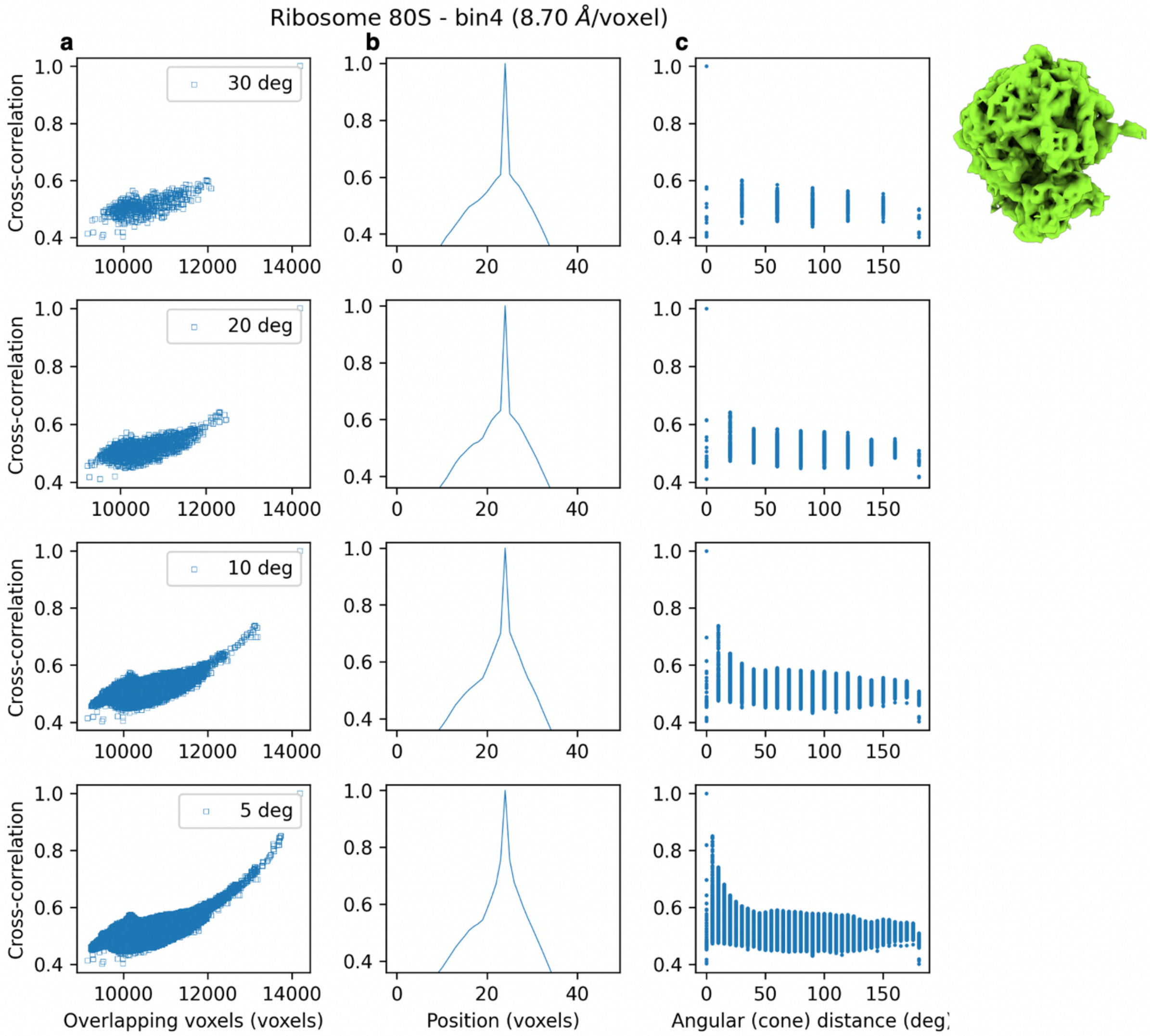

Supplementary Fig. 6: **Evaluation of increasing the number of orientations in template matching of ribosome 80S using the python tool for *in silico* evaluation. a**, Cross-correlation as a function of the absolute number of overlapping voxels for all evaluated rotations. **b**, Cross-correlation as a function of the distance along the z-plane. **c**, Cross-correlation for all evaluated rotations as a function of the angular cone distance. With an increasing number of orientations (decreasing angular sampling), more rotations lead to a higher number of overlapping voxels and thus to a higher cross-correlation. This effect leads to a broadening of the peak in the middle panel.

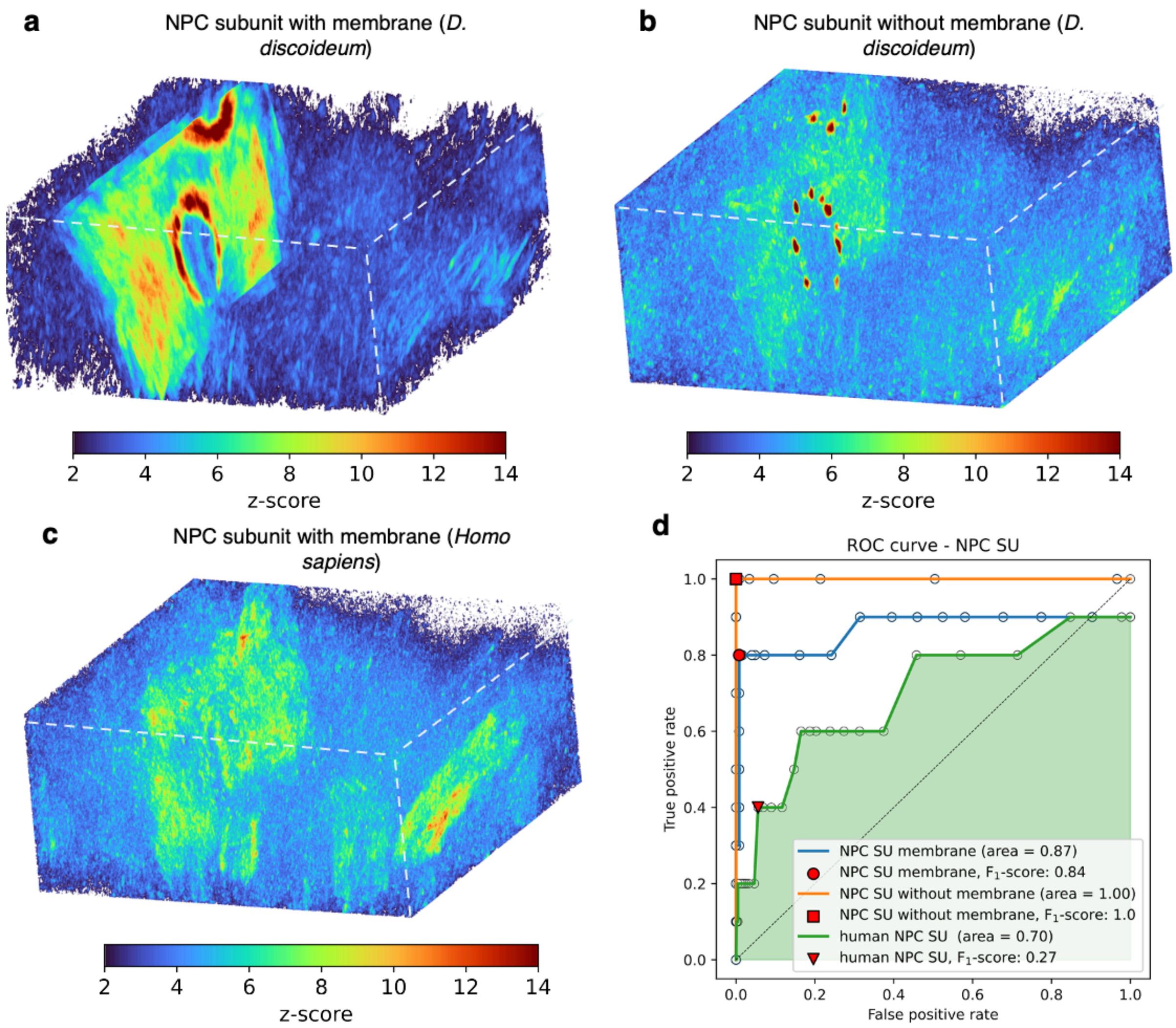

Supplementary Fig. 7: Template matching results for the NPC subunit (C8-symmetric rotational segment) of two different species. Cross correlation (z-score) maps obtained on the same tomographic volume using different templates of the NPC subunit. **a**-**c**, z-scores for NPC subunit templates with (**a**) and without membrane (**b**) for *D. discoideum*, and (**c**) with the NPC template for *Homo sapiens* NPC with membrane[3]. Note that no clear peaks are detected for the *Homo sapiens* NPC subunit, which exemplify the potential of TM for comparing macromolecular complexes of different species. **d,** ROC curves, area under the curve, and maximum F_1-score for the NPC subunits templates.

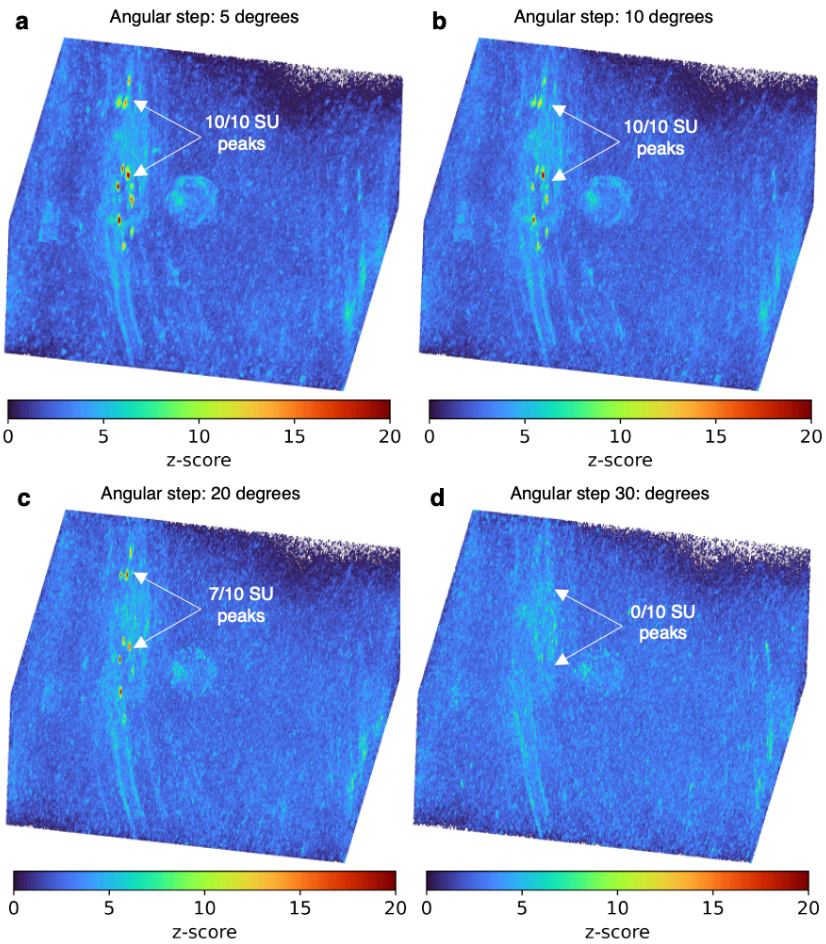

Supplementary Fig. 8: Template matching results for the NPC subunit (C8-symmetric rotational segment) depending on the angular sampling. Cross correlation (z-score) maps obtained on the same tomographic volume (Fig. 1 in the main text) for the NPC subunit (SU) without membrane (D. discoideum) using an angular step of 5 (a), 10 (b), 20 (c) and 30 (d) degrees. Labels indicate the number of visible peaks/total number of subunits in the score map.

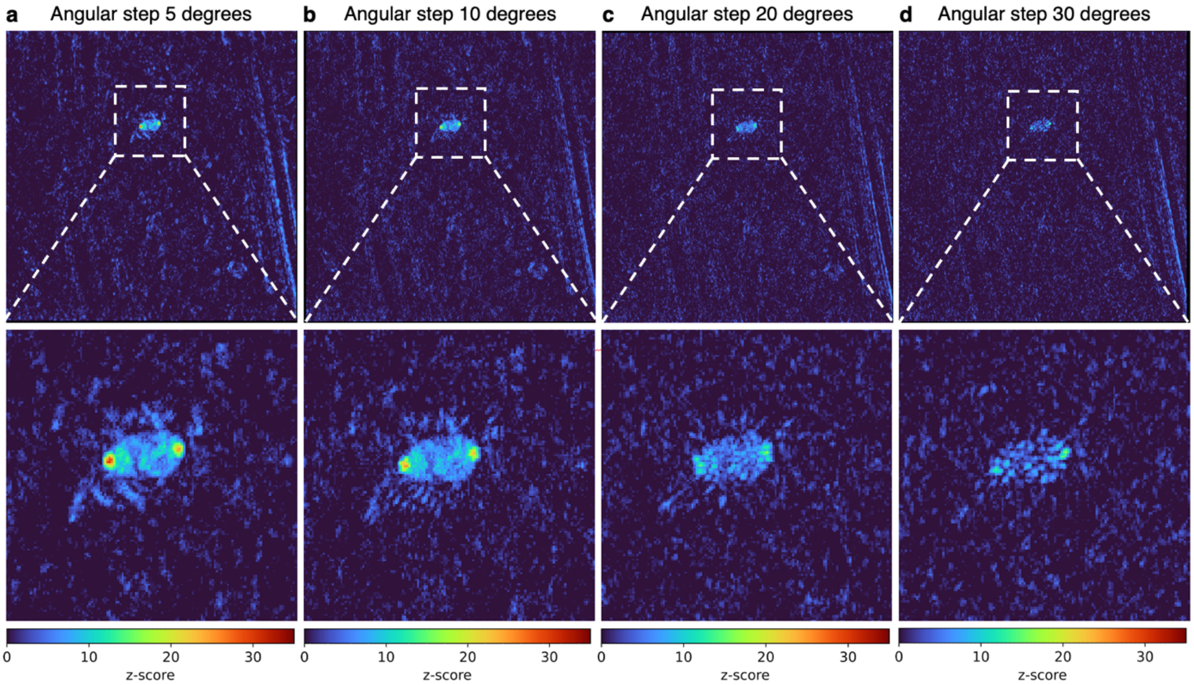

Supplementary Fig. 9: Template matching results for the half vault depending on the angular sampling. Slice of the cross correlation (z-score) maps obtained on the same tomographic volume (Fig. 1 in the main text) for the half vault (*D. discoideum*) using an angular step of 5 (a), 10 (b), 20 (c) and 30 (d) degrees.

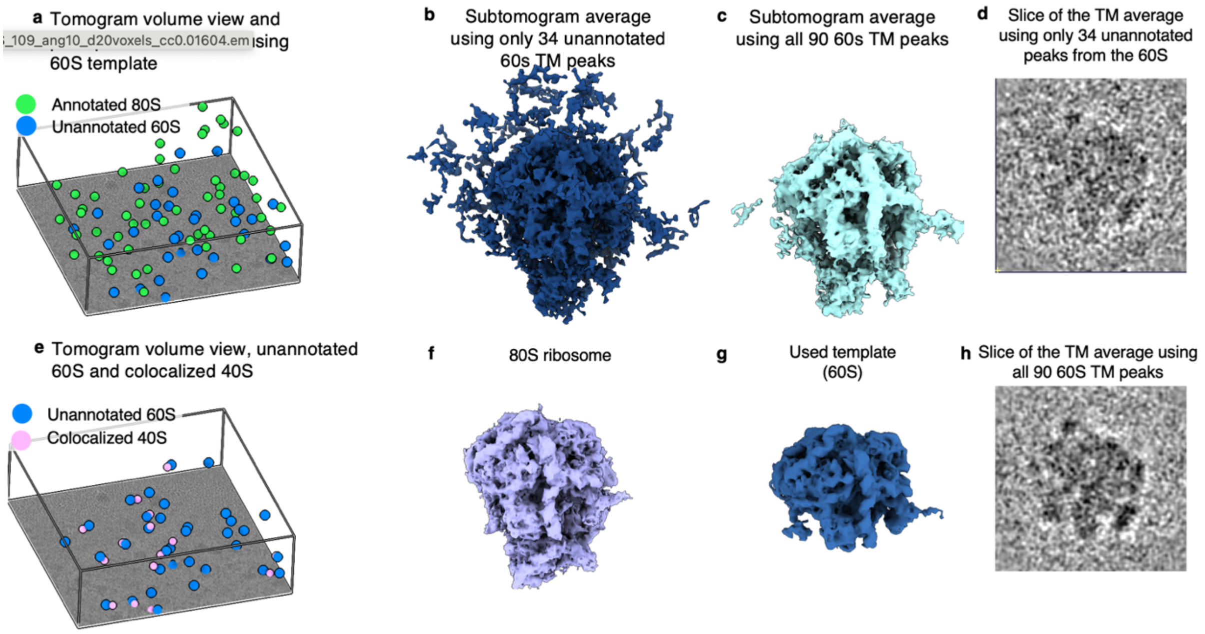

Supplementary Fig. 10: Template matching locates high quality ribosomes. a, Tomogram volume from Fig. 5a, with TM found particles using the 60S template colored as annotated from Relion (green) and unannotated (blue). Structures (**b,c**) and slices (**d,h**) obtained by averaging the particles from TM using the large ribosomal subunit 60S (**g**) as template using only the unannotated particles (**b, d**) and all the TM particles (**c,h**). Note that in both cases, we recover features of the 80S ribosome (**f**). **e,** Tomogram volume as in **a,** for clarity only the the unannotated 60S particles from **a**, and the colocalized peaks using the 40S as template (pink) are shown.

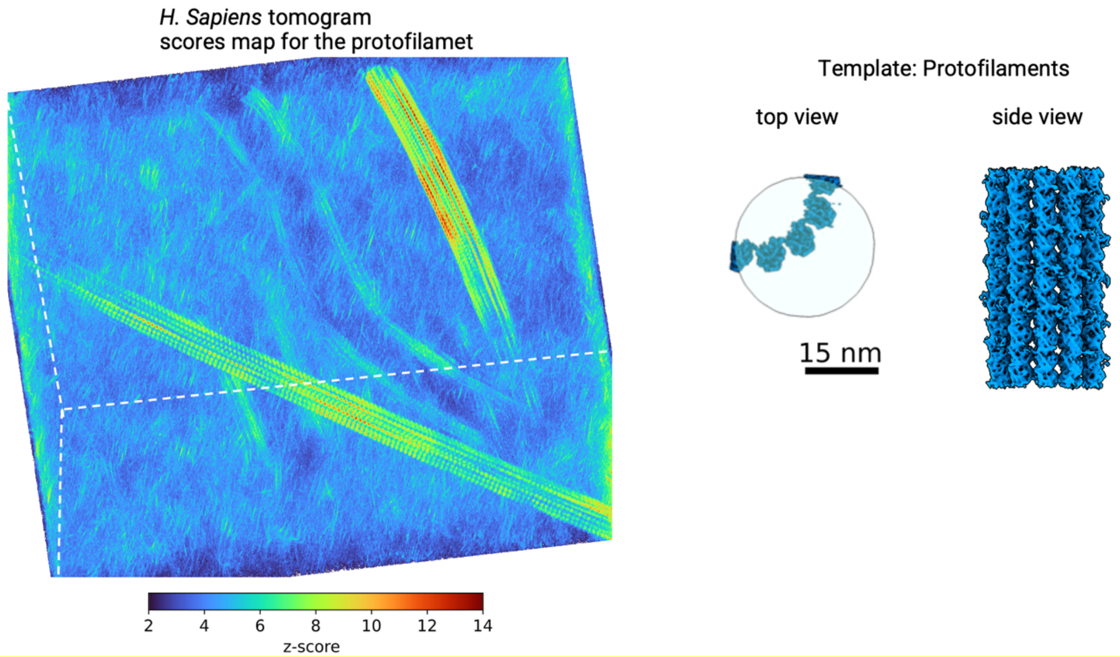

Supplementary Fig. 11: Cross-correlation (z-score) for published tomogram of human tissue culture cells (EMPIAR-11538)[4] The template (right) is a fraction of a microtubule using a cylindrical mask (top view) containing ~4 microtubule protofilaments (three and two halves).

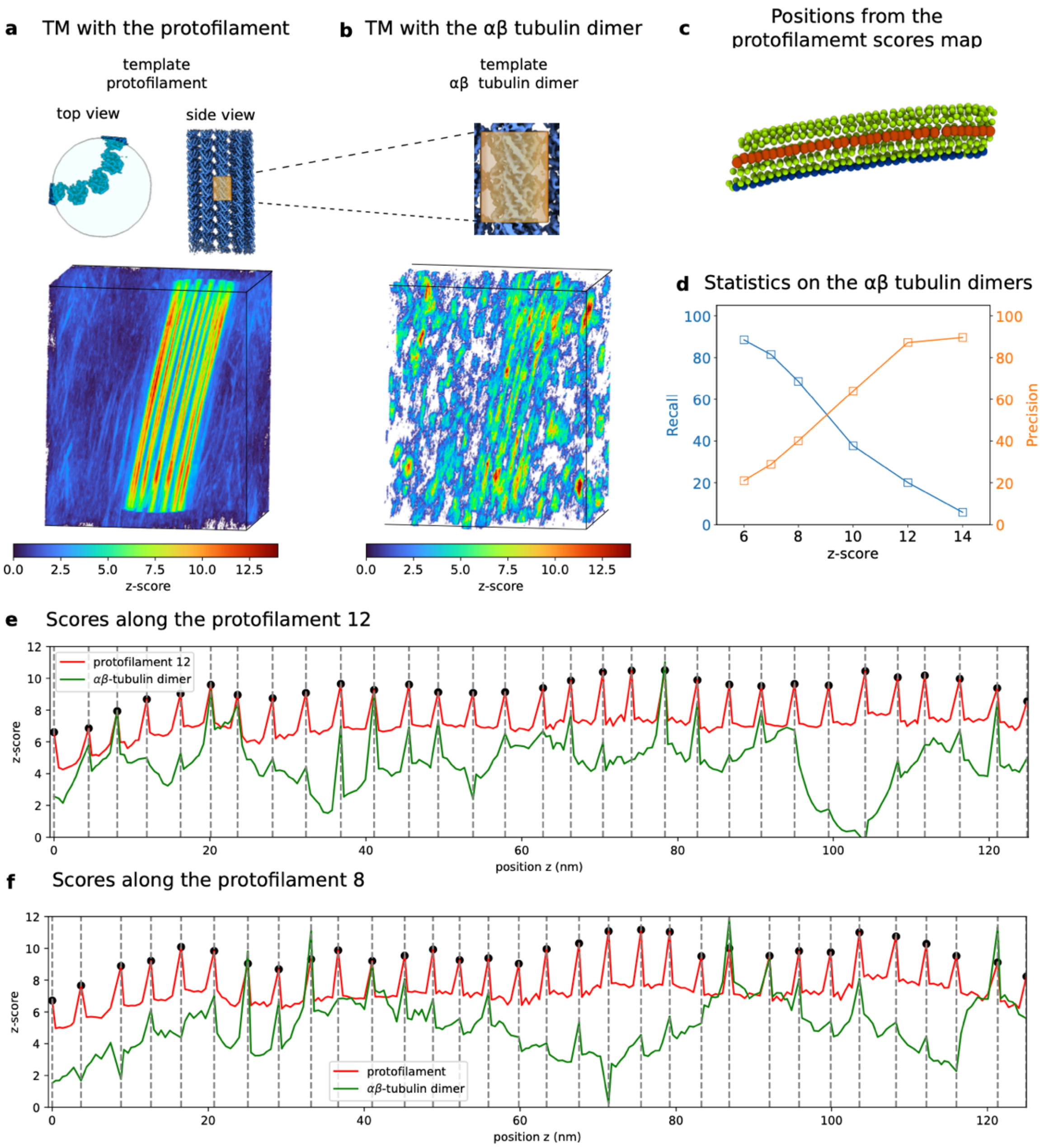

Supplementary Fig. 12: Statistics for template matching using a single ~100 kDa αβ-tubulin dimer. a,**b**, Cross correlation (z-score) maps obtained on the same tomographic volume using a template and a cylindrical mask (top view) containing ~4 microtubule protofilaments (three and two halves) (**a**) and a single αβ-tubulin dimer (**b**). Note that the templates and scores maps on **a** and **b** are identical to Fig. 6**b** in the main text. **c**, Positions of the individual subunits extracted from (**a**), and adopted as annotated particles. Particles colored in brown and blue correspond to protofilaments 12 and 8 as as labelled in Fig 6b used for analysis in panels **e** and **f**. **d**, Statistical analysis of the αβ-tubulin dimer localizations in the volume shown in **b** with the positions in **c** as reference. Shown are the recall = #(true positives) / [#(true positives) + #(false negatives)] (blue, left axis) and the precision = #(true positives) / [#(true positives) + #(false positives)]) (orange, right axis) as functions of the threshold applied to the tubulin dimer z-score. A position was considered as true positive if it was within 3 nm of a position in (**c**). **e**,**f**, z-scores along the axial lines passing through protofilament 12 for the protofilament template (**a**) and the αβ-tubulin dimer template (**b**)**.** The black circles and vertical lines indicate the positions of the extracted peaks in panel **c**.

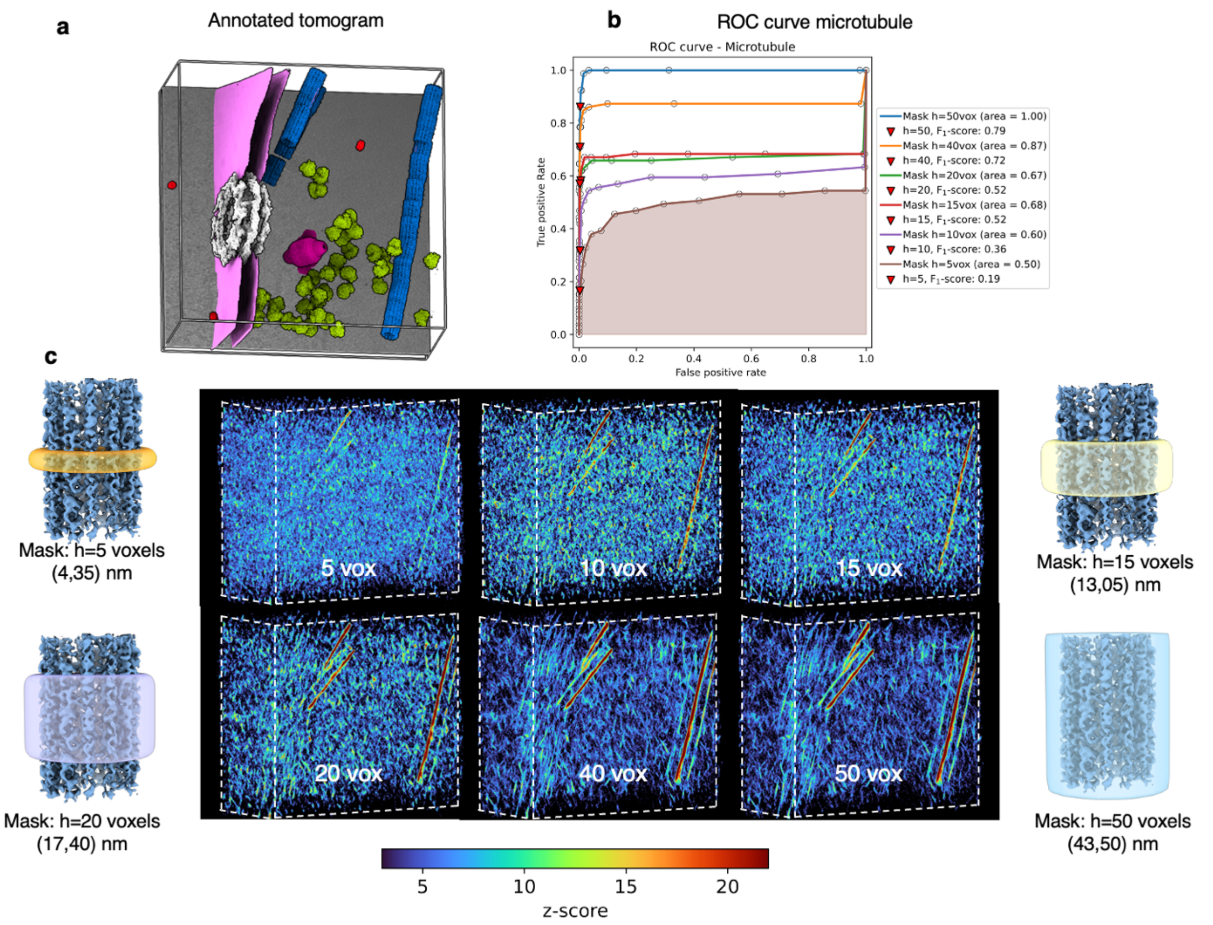

Supplementary Fig. 13: Effect of the effective template size on matching. a, Snapshot of the annotated tomogram used for TM, identical to Fig 1 in the main text. **b**, ROC curves, area under the curve, and maximum F1-score for the microtubule at bin4 with decreasing mask height (h). **c,** Templates and z-scores for microtubules of different height. The left and right panels show the microtubule template (center) with masks of different heights from 5 to 50 voxels (transparent outline). Note that only the fraction of the template within the mask is used for TM. The middle panels show a projection view of the z-score maps obtained with the different masks, with mask heights indicated. Decreasing the height of the masks, i.e., the size of the template used, increases the background noise while decreasing the strength of the microtubule peaks.

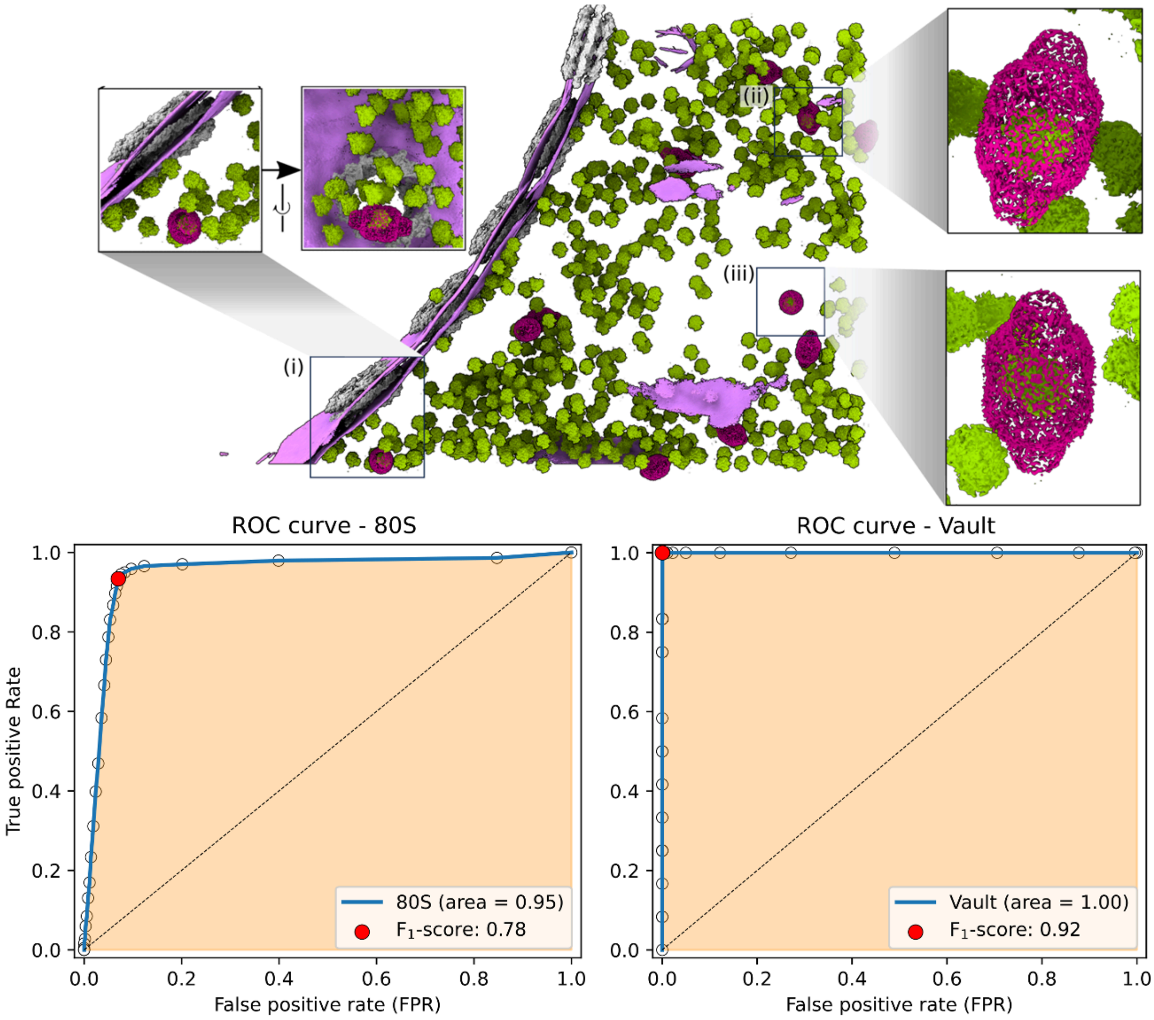

Supplementary Fig. 14: ROC curves for the 80S ribosomes and vaults. a, Identical to Fig. 6**c** in the main text. TM finds ribosomes inside vaults indicated by the squares (i, ii and iii clockwise from left). Note that the templates used for the ribosome and the vault are identical to those used in Fig. 1. Here the vault is shown with a lower threshold level only to visualize the ribosome on the inside. **b,c** show the performance of TM at all classification thresholds using the ROC curves, area under the curve, and maximum F1-score for the 80S (**b**) and the vault (**c**).

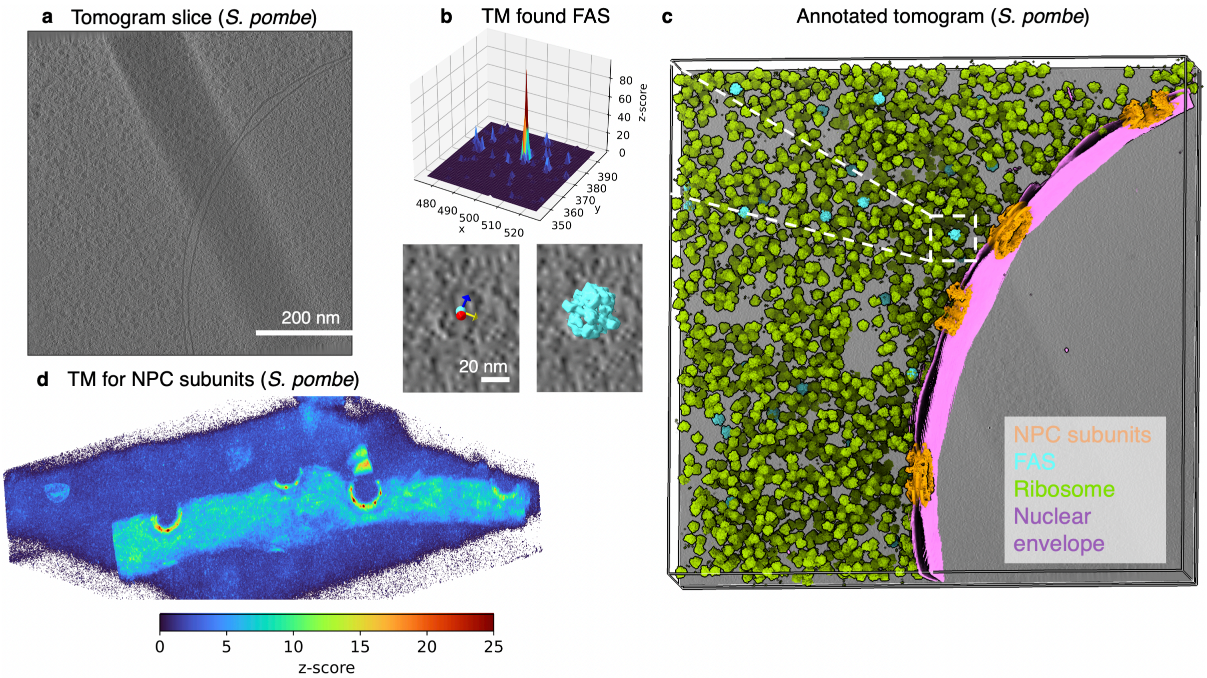

Supplementary Fig. 15: TM results for published tomogram of *S. pombe*. a, Slice of the reconstructed tomogram for *S. pombe* obtained from the publicly available tilt series (EMPIAR-10989). The tomogram in (**a**) is cross-correlated independently with templates of the 80S ribosome (EMD-14426)[5], FAS (EMD-14412)[5], and a subunit of the NPC (C8-symmetric rotational segment) produced from the whole NPC map (EMD-11373)[6]. **b**, Zoomed views of the tomogram at a position identified by TM (top), showing that the peaks correspond to FAS (bottom). **c**, 3D segmentation generated from the z-score maps for visualization[7] combining all tested templates. **d**, Cross-correlation (z-score) maps for *S. pombe* NPC subunit as template.

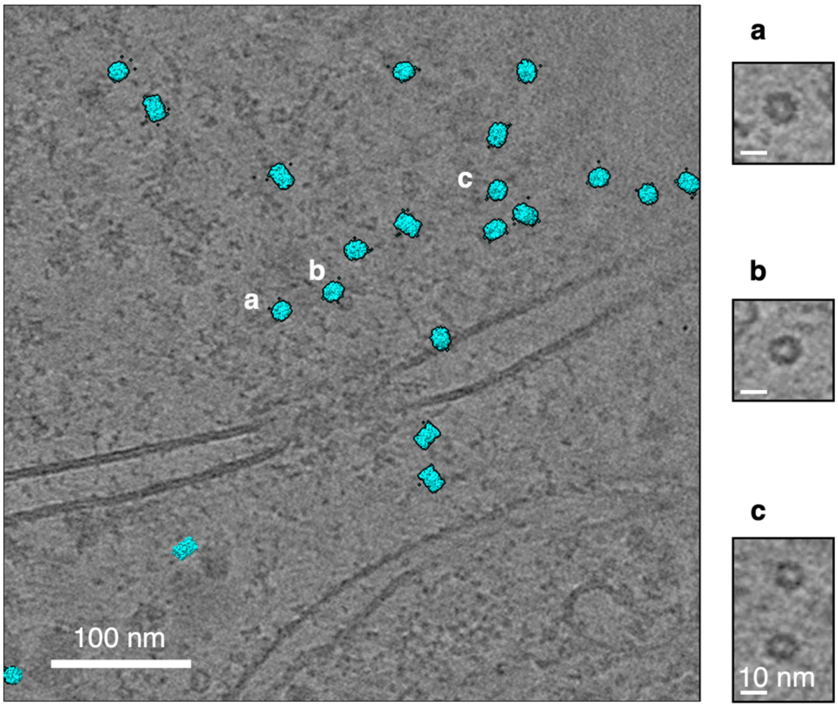

Supplementary Fig. 16: Template matching results for proteasome 20S. Templates (cyan) are repositioned to the position where TM reported high confidence peaks. **a**-**c**, Zoomed views of the tomogram at the positions identified by TM, showing that the peaks correspond to proteasomes.

Supplementary Table 1: Cryo-EM data collection.

	In situ vault (vault) STA[8]
**Data collection and processing**	
Microscope	Titan Krios G2+ FEG
Magnification	42000
Voltage (kV)	300
Electron exposure (e–/Å^2)	~132-150
Defocus range (μm)	-2.5 – 5.0
Pixel size (Å)	2.176
Symmetry imposed	1
Initial particle (no.)	654
Final particle (no.)	654
Map resolution (Å)	29
FSC threshold	0.5

Supplementary References

1. Zivanov, J. *et al.* New tools for automated high-resolution cryo-EM structure determination in RELION-3. *eLife* **7**, e42166 (2018).
2. Hoffmann, P. C. *et al.* Structures of the eukaryotic ribosome and its translational states in situ. *Nat. Commun.* **13**, 7435 (2022).
3. Mosalaganti, S. *et al.* AI-based structure prediction empowers integrative structural analysis of human nuclear pores. *Science* **376**, eabm9506 (2022).
4. Xing, H. *et al.* Translation dynamics in human cells visualized at high resolution reveal cancer drug action. *Science* **381**, 70–75 (2023).
5. de Teresa-Trueba, I. *et al.* Convolutional networks for supervised mining of molecular patterns within cellular context. *Nat. Methods* **20**, 284–294 (2023).
6. Zimmerli, C. E. *et al.* Nuclear pores dilate and constrict in cellulo. *Science* **374**, eabd9776 (2021).
7. Ermel, U. H., Arghittu, S. M. & Frangakis, A. S. ArtiaX : An electron tomography toolbox for the interactive handling of s ub-tomograms in UCSF ChimeraX. *Protein Sci.* **31**, (2022).
8. Cruz-León, S. *et al.* Data for High-confidence 3D template matching for cryo-electron tomography. [Data Set] https://doi.org/10.5281/ZENODO.10819130 (2024).
