# Supplementary material for: High-confidence 3D template matching for cryo-electron tomography
Source: Nat Commun. 2024 May 11;15:3992. doi: 10.1038/s41467-024-47839-8 (PMC11088655; doi:10.1038/s41467-024-47839-8)
Supplement: Supplementary file 3 — Description of Additional Supplementary Files [file 41467_2024_47839_MOESM3_ESM.pdf]

File name: Supplementary Movie 1

Description: Template matching for membrane segmentation. The video shows the cross-correlation maps obtained by high-confidence template matching superimposed on the tomogram. The cross-correlation was obtained for a membrane template (large STA) with an angular sampling of 2 degrees. The high confidence peaks are from the nuclear envelope (see also Fig. 3).
