# Supplementary material for: High-confidence 3D template matching for cryo-electron tomography
Source: Nat Commun. 2024 May 11;15:3992. doi: 10.1038/s41467-024-47839-8 (PMC11088655; doi:10.1038/s41467-024-47839-8)
Supplement: Supplementary file 5 — Reporting Summary [file 41467_2024_47839_MOESM5_ESM.pdf]

nature portfolio

Corresponding author(s): Martin Beck, Beata Turonova and Gerhard Hummer

Last updated by author(s): Jun 29, 2023

Reporting Summary

Nature Portfolio wishes to improve the reproducibility of the work that we publish. This form provides structure for consistency and transparency in reporting. For further information on Nature Portfolio policies, see our Editorial Policies and the Editorial Policy Checklist.

Statistics

For all statistical analyses, confirm that the following items are present in the figure legend, table legend, main text, or Methods section.

n/a	Confirmed	
☐	☒	The exact sample size (n) for each experimental group/condition, given as a discrete number and unit of measurement
☐	☒	A statement on whether measurements were taken from distinct samples or whether the same sample was measured repeatedly
☒	☐	The statistical test(s) used AND whether they are one- or two-sided<br>*Only common tests should be described solely by name; describe more complex techniques in the Methods section.*
☒	☐	A description of all covariates tested
☒	☐	A description of any assumptions or corrections, such as tests of normality and adjustment for multiple comparisons
☐	☒	A full description of the statistical parameters including central tendency (e.g. means) or other basic estimates (e.g. regression coefficient) AND variation (e.g. standard deviation) or associated estimates of uncertainty (e.g. confidence intervals)
☒	☐	For null hypothesis testing, the test statistic (e.g. F, t, r) with confidence intervals, effect sizes, degrees of freedom and P value noted<br>*Give P values as exact values whenever suitable.*
☒	☐	For Bayesian analysis, information on the choice of priors and Markov chain Monte Carlo settings
☒	☐	For hierarchical and complex designs, identification of the appropriate level for tests and full reporting of outcomes
☒	☐	Estimates of effect sizes (e.g. Cohen's d, Pearson's r), indicating how they were calculated

Our web collection on statistics for biologists contains articles on many of the points above.

Software and code

Policy information about availability of computer code

Data collection	The software used for data collection was SerialEM (versions 3.8.1) (see Methods section of the manuscript and the original publication where the data were collected: Hoffmann, P.C., Kreysing, J.P., Khusainov, I. et al. Structures of the eukaryotic ribosome and its translational states in situ. Nat Commun 13, 7435 (2022)).
Data analysis	All code developed for this study is part of the public repositories.<br>GAPSTOPTM is available at https://gitlab.mpcdf.mpg.de/bturo/gapstop_tm. Documentation and installation instructions are provided for ease of use (https://bturo.pages.mpcdf.de/gapstop_tm).<br><br>In silico peak analysis: The in silico peak analysis is part of the Contextual Analysis Tools for CryoET and subtomogram averaging (cryoCAT). The source code of cryoCAT is available in the following repository: https://github.com/turonova/cryoCAT. A detailed notebook outlining the parameters and usage of the in silico peak analysis can be found here: https://github.com/turonova/cryoCAT/blob/main/docs/source/tutorials/peak_analysis/peak_analysis.ipynb<br><br>Existing software used in this study is described in the Methods section of the manuscript.<br>STOPGAP (https://github.com/williamnwan/STOPGAP), IMOD (versions 4.10.9 and 4.11.5), ChimeraX (versions 1.4 and 1.5), eman2 (https://blake.bcm.edu/emanwiki/EMAN2), Matlab R2019b, SWISS-MODEL (https://swissmodel.expasy.org), python (version 3.9).

For manuscripts utilizing custom algorithms or software that are central to the research but not yet described in published literature, software must be made available to editors and reviewers. We strongly encourage code deposition in a community repository (e.g. GitHub). See the Nature Portfolio guidelines for submitting code & software for further information.

Data

Policy information about availability of data

All manuscripts must include a data availability statement. This statement should provide the following information, where applicable:

- Accession codes, unique identifiers, or web links for publicly available datasets
- A description of any restrictions on data availability
- For clinical datasets or third party data, please ensure that the statement adheres to our policy

The previously published structures for the NPC subunits (H. sapiens) EMD-14325, EMD-14328 and EMD-14330, the NPC (S. pombe) EMD-11373, the 80S ribosome (D. discoideum) EMD-15810, EMD-15812, and EMD-15815, the 80S ribosome (S. pombe) EMD14426, the fatty acid synthase (S. pombe) EMD-14412, the 20S proteasome (H. sapiens) EMD-4877 and the microtubule (H. sapiens) EMD-6351 are accessible through the Electron Microscopy Data Bank. The tilt series of D. discoideum were previously reported (codes: EMPIAR-11845 and EMPIAR-11899). The previously published tilt series for S. pombe, EMPIAR-10989 and Hek293 cells (EMPIAR-11538) are available through the Electron Microscopy Public Image Archive. The previously published structures 7R5J, 6RGQ, and 3JAR are available through the Protein Data Base. Source data are provided with this paper. Molecular dynamics setups, templates generated in this study and supplementary raw data have been deposited in Zenodo (https://doi.org/10.5281/zenodo.10819130).

Research involving human participants, their data, or biological material

Policy information about studies with human participants or human data. See also policy information about sex, gender (identity/presentation), and sexual orientation and race, ethnicity and racism.

Reporting on sex and gender	Study does not involve human research participants.
Reporting on race, ethnicity, or other socially relevant groupings	Study does not involve human research participants.
Population characteristics	Study does not involve human research participants.
Recruitment	Study does not involve human research participants.
Ethics oversight	Study does not involve human research participants.

Note that full information on the approval of the study protocol must also be provided in the manuscript.

Field-specific reporting

Please select the one below that is the best fit for your research. If you are not sure, read the appropriate sections before making your selection.

☒ Life sciences ☐ Behavioural & social sciences ☐ Ecological, evolutionary & environmental sciences

For a reference copy of the document with all sections, see nature.com/documents/nr-reporting-summary-flat.pdf

Life sciences study design

All studies must disclose on these points even when the disclosure is negative.

Sample size	We have not predetermined the sample size. We analyze the maximum amount of data for the computational resources available.
Data exclusions	We include all analyzed data.
Replication	A total of 32 different tomograms with up to 21 templates were successfully analyzed. Each computation and analysis was independent.
Randomization	N.A.
Blinding	N.A.

Reporting for specific materials, systems and methods

We require information from authors about some types of materials, experimental systems and methods used in many studies. Here, indicate whether each material, system or method listed is relevant to your study. If you are not sure if a list item applies to your research, read the appropriate section before selecting a response.

Materials & experimental systems

n/a	Involved in the study
☒	☐ Antibodies
☐	☒ Eukaryotic cell lines
☒	☐ Palaeontology and archaeology
☒	☐ Animals and other organisms
☒	☐ Clinical data
☒	☐ Dual use research of concern
☒	☐ Plants

Methods

n/a	Involved in the study
☒	☐ ChIP-seq
☒	☐ Flow cytometry
☒	☐ MRI-based neuroimaging

Eukaryotic cell lines

Policy information about cell lines and Sex and Gender in Research

Cell line source(s)	The D. discoideum cell line (GFP-Nup62) used in this study was derived from the Ax2-214 strain of the Gerisch Lab (MPI Martinsried, Germany). The Ax2-214 strain is available through dyctybase. See also the original publication where the data were collected: Hoffmann, P.C., Kreysing, J.P., Khusainov, I. et al. Structures of the eukaryotic ribosome and its translational states in situ. Nat Commun 13, 7435 (2022).
Authentication	The D. discoideum cell line used in this study was not authenticated.
Mycoplasma contamination	Cell lines were not tested for mycoplasma contamination, since it is not relevant to D. discoideum.
Commonly misidentified lines (See ICLAC register)	None
