# Supplementary material for: High-confidence 3D template matching for cryo-electron tomography
Source: Nat Commun. 2024 May 11;15:3992. doi: 10.1038/s41467-024-47839-8 (PMC11088655; doi:10.1038/s41467-024-47839-8)

# npc (su_avg_masked) matched with npc (su_resampled)

| | |
|---|---|
| Symmetry | 1 |
| Apply wedge | False |
| Degrees | 20 |
| Apply angular offset | False |
| Binning | 4 |
| Pixelsize | 8.704 |
| Boxsize | 124 |
| Voxels | 119540.0 |
| Voxels TM | 45348.0 |
| Solidity | 0.2161 |
| Dimensions | [59. 98. 82.] |

| | |
|---|---|
| Peak value | 0.598 |
| Peak center | [62. 61. 62.] |
| Drop | [0.0456 0.0258 0.0593] |
| Mean | [0.5607 0.5042 0.4082 0.3447 0.2978] |
| Median | [0.5544 0.4974 0.3959 0.3306 0.2756] |
| Var | [0.0007 0.0024 0.0066 0.0085 0.0072] |

| | |
|---|---|
| Dist maps Solidity | [0.4599 0.3297 0.5876] |
| Dist maps VC | [ 9371. 36339. 1120322.] |
| Dist maps VC open | [ 8810. 33163. 1040113.] |
| Open dist_all | [40. 41. 27.] |
| Open dist_normals | [77. 88. 27.] |
| Open dist_inplane | [124. 124. 124.] |

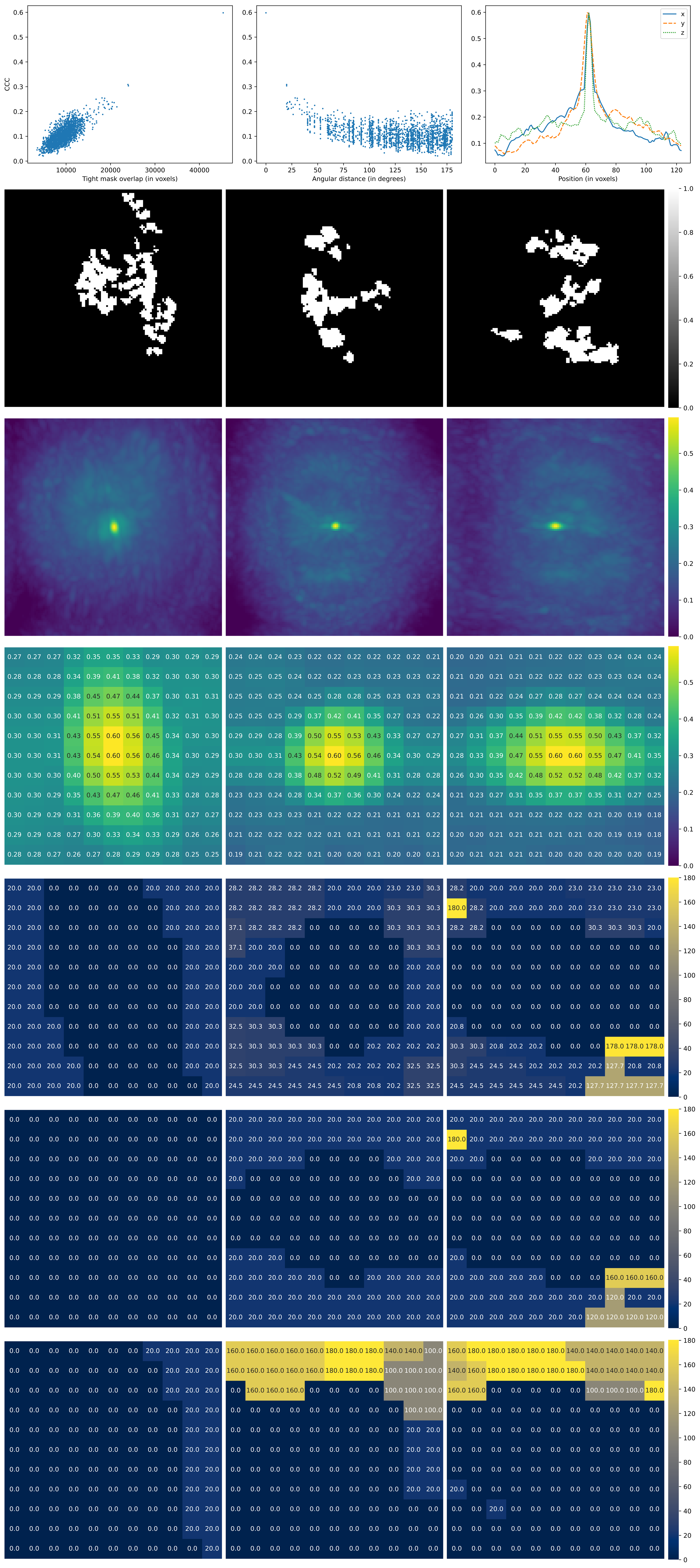

Supplement: Supplementary file 6 — Source Data [file 41467_2024_47839_MOESM6_ESM.zip › Source_data_file/Supplementary_Figures/Supplementary_Fig4/In_silico_NPC_SU_results/id_71_summary.pdf]
