# Supplementary material for: High-confidence 3D template matching for cryo-electron tomography
Source: Nat Commun. 2024 May 11;15:3992. doi: 10.1038/s41467-024-47839-8 (PMC11088655; doi:10.1038/s41467-024-47839-8)

vault (bin4_voxel2.176_vault_STA_tomsa_symmetrized) matched with self

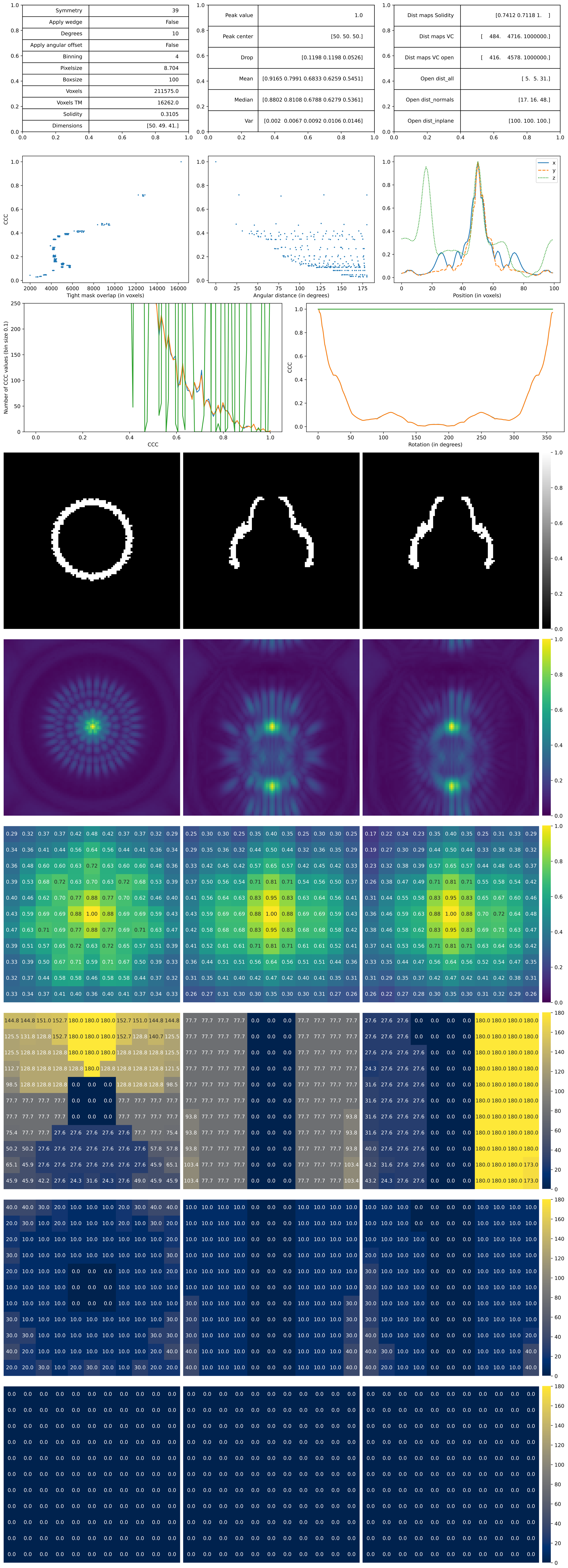

Supplement: Supplementary file 6 — Source Data [file 41467_2024_47839_MOESM6_ESM.zip › Source_data_file/Supplementary_Figures/Supplementary_Fig2/In_silico_Vault_results/id_3_summary.pdf]
