# Supplementary figures and images for: High-confidence 3D template matching for cryo-electron tomography
Source: Nat Commun. 2024 May 11;15:3992. doi: 10.1038/s41467-024-47839-8 (PMC11088655; doi:10.1038/s41467-024-47839-8)

membrane (bin4\_voxel2.176\_membrane\_STA\_50voxel\_symmetrized) matched with self

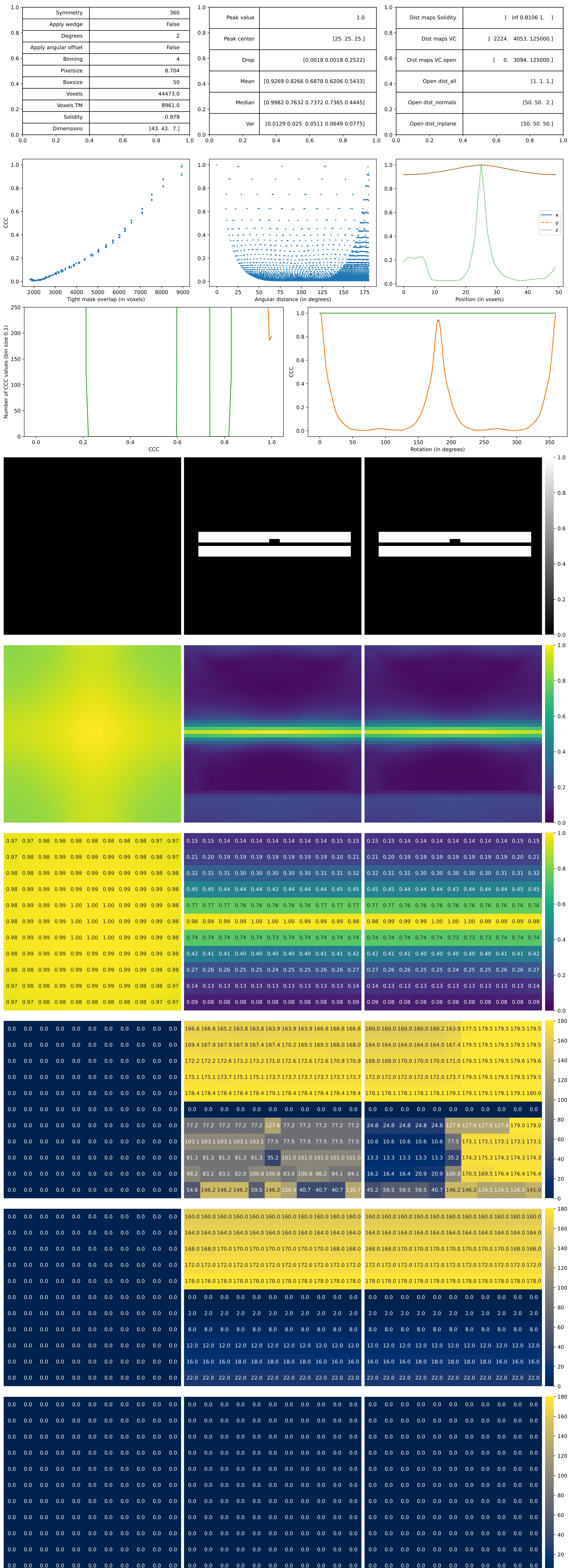

Supplement: Supplementary file 6 — Source Data [file 41467_2024_47839_MOESM6_ESM.zip › Source_data_file/Supplementary_Figures/Supplementary_Fig3/In_silico_membrane_results/id_29_summary.pdf]

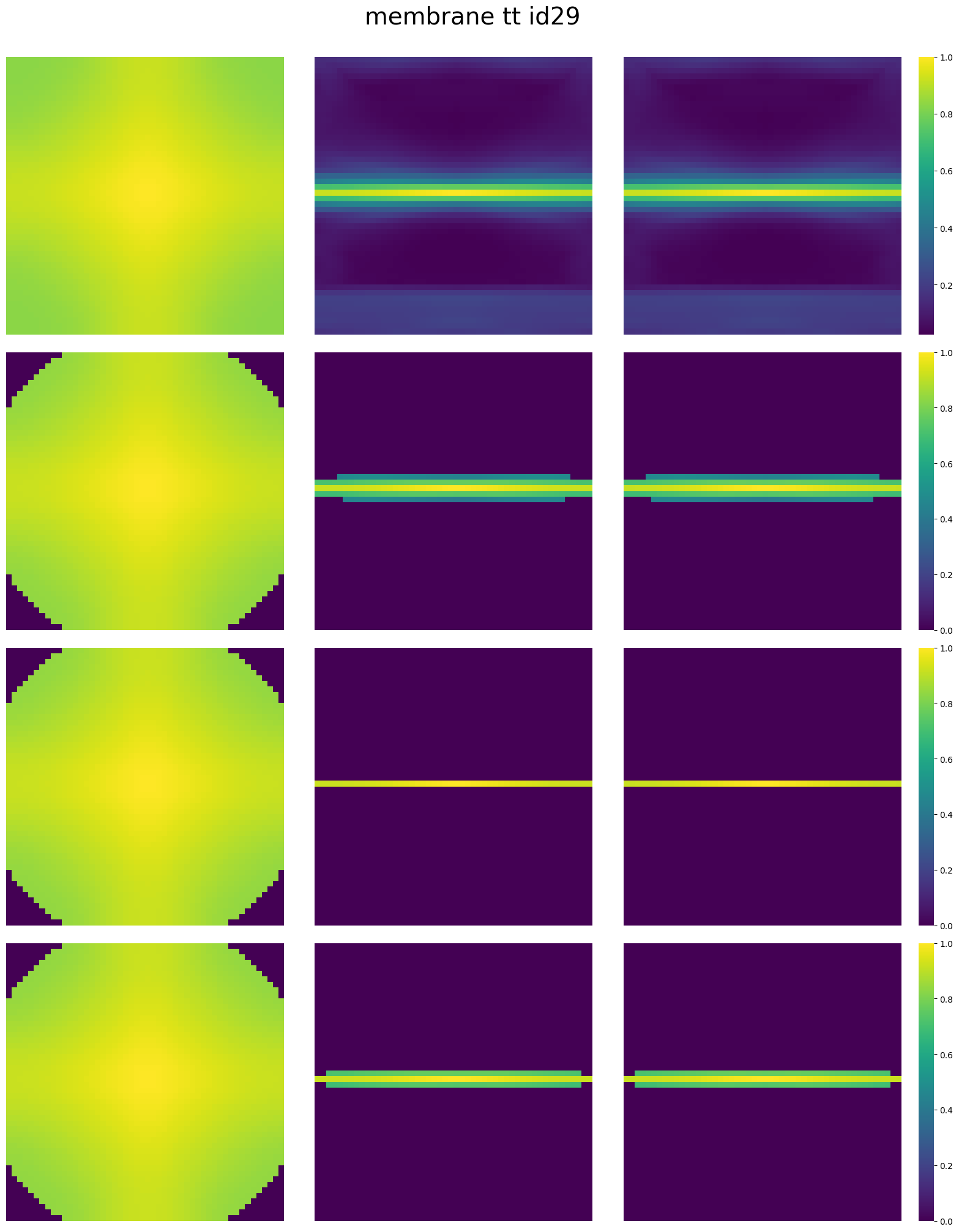

Supplement: Supplementary file 6 — Source Data [file 41467_2024_47839_MOESM6_ESM.zip › Source_data_file/Supplementary_Figures/Supplementary_Fig3/In_silico_membrane_results/peaks.png]
